# Understanding the molecular mechanism of Ginkgo Folium-Forsythiae Fructus for cerebral atherosclerosis treatment using network pharmacology and molecular docking

**DOI:** 10.1097/MD.0000000000032823

**Published:** 2023-02-17

**Authors:** Jinfei Zhang, Jialin Gai, Hengqin Ma, Jiqin Tang, Chuntao Yang, Guoxiu Zu

**Affiliations:** a Department of Rehabilitation Medicine, Shandong University of Traditional Chinese Medicine, Jinan, China; b Hospital Management Office, Shandong University of Traditional Chinese Medicine, Jinan, China; c Department of Traditional Chinese Medicine, Shandong University of Traditional Chinese Medicine, Jinan, China.

**Keywords:** cerebral atherosclerosis, Forsythiae Fructus, Ginkgo Folium, mechanism, network pharmacology

## Abstract

Cerebral atherosclerosis (CA) is a chronic disease caused by multiple infarcts and atrophy causing nerve degenerative syndrome. Ginkgo Folium (GF) and Forsythiae Fructus (FF) have shown positive effects on vascular protection, but their relationship with CA is unclear. This study aimed to identify the potential CA targets and mechanisms of action of GF-FF, using network pharmacology.

**Objective:**

This study used network pharmacology and molecular docking to examine the potential targets and pharmacological mechanism of GF-FF on CA.

**Methods:**

Using the traditional Chinese medicine systems pharmacology database and analysis platform, components were screened and corresponding targets were predicted using boundary values and Swiss Target Prediction. Using Cytoscape 3.8.0, a network was established between GF-FF components and CA targets. We extracted disease genes and constructed a network of targets based on the protein-protein interaction networks functional enrichment analysis database. Using Metascape, the Gene Ontology and the Kyoto Encyclopedia of Genes and Genomes of the enriched targets were determined. AutoDock Vina was used to perform molecular docking.

**Results:**

Twenty-three active ingredients of GF-FF were confirmed to treat CA, covering 109 targets, of which 48 were CA-related. Luteolin, bicuculline, sesamin, kaempferol, quercetin, and ginkgolide B were the vital active compounds, and EGFR, CYP2E1, CREB1, CYP19A1, PTGS2, PPARG, PPARA, ESR1, MMP9, MAPK14, MAPK8, and PLG were the major targets. The molecular docking showed that these compounds and targets exhibited good intercalation. These 48 protein targets produced effects on CA by modulating pathways such as “apoptosis–multiple species,” “IL-17 signaling pathway,” and “relaxin signaling pathway.”

**Conclusions:**

As predicted by network pharmacology, GF-FF exerts anti-tumor effects through multiple components and targets for treatment of CA, providing new clinical ideas for CA treatment.

## 1. Introduction

Cerebral atherosclerosis (CA) is a highly prevalent cerebrovascular disease with frequent subclinical and clinical ischemia.^[1]^ Numerous experiments have confirmed racial differences in CA, and CA is particularly prevalent in Asian, Black, Hispanic, and Indian populations. In the Chinese population, the incidence of CA is up to 46.6% in stroke patients.^[2–4]^

The development of CA may be associated with a range of risk factors, such as age, hypertension, diabetes, hyperlipidemia, metabolic syndrome, obesity, and smoking.^[5,6]^ Current treatment for CA includes anti-platelet drugs (aspirin, warfarin,^[5–7]^ ticagrelor,^[7,8]^ and cilostazol),^[6]^ lipid-regulating drugs (atorvastatin, angiotensin-converting enzyme inhibitors),^[5,9]^ and surgical treatment (reconstruction of the vessel wall,^[5]^ placement of arterial stents).^[8,10]^ These treatments can treat the symptoms of CA, but accordingly lead to many clinical adverse events.^[5,11]^ Therefore, traditional Chinese medicine (TCM) is a complementary or alternative approach in the treatment of CA.

Chinese medicine has been widely used in the clinical treatment of CA.^[12]^ Ginkgo Folium (GF) is sweet, bitter, astringent, and flat, and according to the Chinese Medicine Journal,^[13]^ it can be “astringent to lung qi, stop turbidity, and calm cough and asthma.” Forsythiae Fructus (FF) is bitter and slightly cold and was first described in detail in the classic of Materia medica.^[14]^ GF-FF has a wide range of effects, including anti-inflammatory, antioxidant, hypolipidemic,^[15]^ anti-platelet aggregation, and vascular protection effects.^[16]^ Several studies have shown that sesamin and ginkgolide B, as components of the woody component and terpenoids of GF, respectively, have significant effects on platelet antagonism and vascular protection,^[17,18]^ their main constituents are listed in Table 1. Quercetin, kaempferol, and luteolin, as Chinese herbal ingredients of FF, mainly exhibit anti-inflammatory, antioxidant, and antibacterial effects,^[19,20]^ and inhibit cancer cell production and apoptosis. However, the mechanism of therapeutic action of GF-FF in the treatment of CA is unclear, and the potential anti-CA mechanism of GF-FF must be elucidated.

**Table 1:** Information for candidate active compounds for GF, FF.

Number	Compound	OB(%)	DL	GI absorption	herb	CAS
MOL011578	Bilobalide	84.42	0.36	High	GF	33570-04-6
MOL011604	Syringetin	36.82	0.37	High	GF	4423-37-4
MOL001494	Mandenol	42.00	0.19	High	GF	544-35-4
MOL001558	sesamin	56.55	0.83	High	GF	607-80-7
MOL002881	Diosmetin	31.14	0.27	High	GF	520-34-3
MOL003044	Chryseriol	35.85	0.27	High	GF	491-71-4
MOL000354	isorhamnetin	49.60	0.31	High	GF	480-19-3
MOL000492	(+)-catechin(+)-	54.83	0.24	High	GF	154-23-4
MOL005573	Genkwanin	37.13	0.24	High	GF	437-64-9
MOL007179	Linolenic acid ethyl ester	46.10	0.20	High	GF	1191-41-9
MOL000096	(-)-catechin(-)-	49.68	0.24	High	GF	154-23-4
MOL011586	ginkgolide B	44.38	0.73	High	GF	15291-77-7
MOL000173	wogonin	30.68	0.23	High	FF	632-85-9
MOL003283	(2R,3R,4S)-4-(4-hydroxy-3-methoxy-phenyl)-7-methoxy-2,3-dimethylol-tetralin-6-ol	66.51	0.39	High	FF	548-29-8
MOL003290	(3R,4R)-3,4-bis[(3,4-dimethoxyphenyl) methyl] oxolan-2-one	52.30	0.48	High	FF	17238-81-7
MOL003306	ACon1_001697	85.12	0.57	High	FF	487-41-2
MOL003330	(-)-Phillygenin	95.04	0.57	High	FF	487-39-8
MOL003370	Onjixanthone I	79.16	0.30	High	FF	N/A
MOL000422	kaempferol	41.88	0.24	High	GF/FF	520-18-3
MOL000006	luteolin	36.16	0.25	High	GF/FF	491-70-3
MOL000791	bicuculline	69.67	0.88	High	FF	485-49-4
MOL000098	quercetin	46.43	0.28	High	GF/FF	117-39-5
MOL003331	Forsythiaside	3.05	0.61	High	FF	79916-77-1

FF = Forsythiae Fructus, GF = Ginkgo Folium, OB = oral bioavailability.

**Figure 1.**
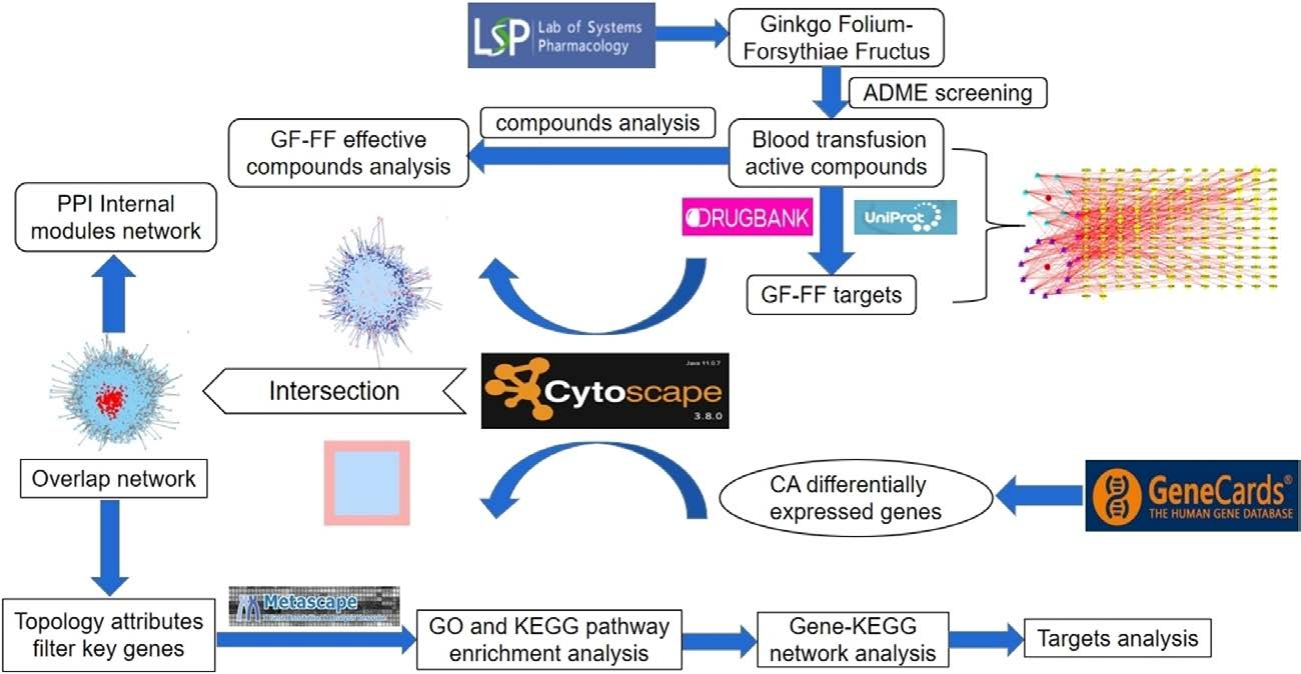
Flowchart of study.

As part of systems biology, network pharmacology combines pharmacology, bioinformatics, and molecular biology, and has been used to illustrate the multi-targeted effects of herbal medicine in a variety of diseases. The aim of this study was to investigate the mechanism of action of GF-FF for the treatment of CA and to predict the target proteins for drug therapy through network pharmacology and molecular docking. The GF-FF treatment CA process is shown in Figure 1.

## 2. Materials and methods

### 2.1. Building a database of compounds

Databases such as CNKI (https://www.cnki.net/), Pubmed (https://pubmed.ncbi.nlm.nih.gov/), Pubchem(https://pubchem.ncbi.nlm.nih.gov/),Medline(https://www.bionity.com/),Wanfang(https://www.wanfangdata.com.cn/), and Web of Science (https://www.webofscience.com/) were searched to identify compounds with a potential effect on CA. The constituents of GF-FF were determined from the (https://old.tcmsp--e.com/tcmsp.php) traditional Chinese medicine systems pharmacology database and analysis platform (TCMSP).^[21]^ The oral bioavailability (OB) and drug-likeness (DL) indices recommended by TCMSP were used to verify the drug-forming properties of each compound. In the TCMSP system, OB ≥ 30% and DL ≥ 0.18 were set as the criteria for screening candidate compounds from GF-FF. High OB and DL may result in better absorption rates in clinical settings.

Swiss absorption, distribution, metabolism, and excretion (ADME) (http://www.swissadme.ch/) is a website that provides molecular pharmacokinetic characterization.^[22]^ It can predict the clinical parameters, pharmacokinetic properties, drug-like properties, and medicinal chemistry friendliness of 1 or more small molecules.^[23,24]^ In this investigation, SwissADME was used to further screen the compound molecules that passed the TCMSP primary screening, with the main screening criteria being that the gastrointestinal absorption was “high.” As a result, a total of 23 GF-FF compounds were identified, in combination with the literature review.

SwissTargetPrediction (http://www.swisstargetprediction.ch/) is a web tool that aims to predict the most probable protein targets of small molecules.^[25,26]^ We import the compounds that meet the criteria after screening into SwissTargetPrediction system for protein target prediction.

### 2.2 . Construction of composite target network

Cytoscape is a software that graphically displays, analyzes, and edits networks, and adds rich annotation information.^[27,28]^ For visualization, GF-FF potential core compounds with corresponding predicted target genes were imported into Cytoscape 3.8.0 to build a compound herbal target gene network. Each compound and target gene in the network is represented by a node, and the relationship between the nodes is represented by a straight line.

### 2.3. Mining for disease targets and target genes

Protein targets related to cerebral atherosclerosis were provided by (https://www.genecards.org, Genecards) with the search term “cerebral atherosclerosis.” Subsequently, the protein data was entered into (https://www.uniprot.org/) (Uniprot) to search to the name of the regulated gene. All target searches were based on “*Homo sapiens*.”

### 2.4. Protein-protein interaction (PPI) network construction

To further investigate the role of the related targets between GF-FF and CA, we input the related gene information into protein-protein interaction networks functional enrichment analysis (STRING) (https://cn.string-db.org/) to analyze the protein-protein interactions. We entered the target genes from the intersection of herbal compounds and diseases into the database, selected the “*Homo sapiens*” biological series, selected “evidence” in the meaning of network edges column, set the confidence to > 0.4, removed the free proteins, obtained the correlation data between the targets, and imported the acquired data into Cytoscape 3.8.0. By combining all the drug and disease target information, the PPI network was constructed, and 12 core protein targets were selected for prediction through network topology analysis.

### 2.5. Gene ontology (GO) enrichment and Kyoto encyclopedia of genes and genomes (KEGG) pathway analysis

To study the biological functions of potential targets in CA, the Metascape (https://metascape.org/) database was used to collect information on GO and KEGG analysis data.^[29]^ The GO enrichment analysis was used to screen molecular functions, biological processes, and cellular components.^[30]^ KEGG analysis was used to describe the regulatory pathway corresponding to the expressed gene. Subsequently, GO and KEGG data were uploaded to the Bioinformatics (http://www.bioinformatics.com.cn/) and imageGP (http://www.ehbio.com/ImageGP/) platforms for visualization and analysis. Based on the enrichment values we imported the top 20 KEGG pathways and core targets into Cytoscape 3.8.0 and used its plug-in ClueGo 2.5.8 to visualize the protein-target-gene pathway network.^[31]^

### 2.6. Validation of compound-target interaction

Molecular docking is a method of drug design which uses interactions between drug molecules to allow prediction of compound and protein binding and affinity patterns.^[32]^ This study used molecular docking to verify the tightness of binding of CA-associated proteins to GF-FF compound molecules, as follows:

1. Core proteins were imported into the protein data bank (https://www.rcsb.org/) and 3D structures were downloaded based on the results of the network pharmacological topology analysis^.[33]^ Protein molecules were imported into AutoDock Tools (version 1.5.6 https://autodock.scripps.edu/) software to remove water molecules, hydrogen atoms were added and protein types were set, and finally, the protein data bank, partial charge and atom type files were saved.
2. Drug molecular structures were obtained from TCMSP and PubChem (https://pubchem.ncbi.nlm.nih.gov/) databases^.[34]^ Energy minimization of compounds was done using Chem3D, saving as mol2 format. Similarly, water molecules were removed, hydrogen atoms were added, drugs were set as ligands in AutoDock Tools, all flexible bonds were made rotatable by default, and finally, the protein data bank, partial charge and atom type file was saved.
3. All the treated compounds were used as small molecule ligands and 12 important protein targets were used as receptors. The center position of the grid box was determined according to the interaction between small molecules and targets, and the length, width, and height were set to 50 × 50 × 50. The files were subsequently imported into AutoDock Vina to perform molecular docking, and the results of GF-FF compounds with core protein targets were visualized using python molecule 2.1 (https://pymol.org/2/)^.[35]^

## 3. Results

### 3.1. Compound-target network construction

We obtained a total of 23 compounds after TCMSP and SwissADME screening of the GF-FF active compounds from the TCMSP database search and literature review. Since Forsythiaside is easily absorbed by the gastrointestinal tract and has significant neuroprotective, anti-inflammatory, and antioxidant effects,^[36,37]^ we also included it in our study, as shown in Table 1.

**Figure 2.**
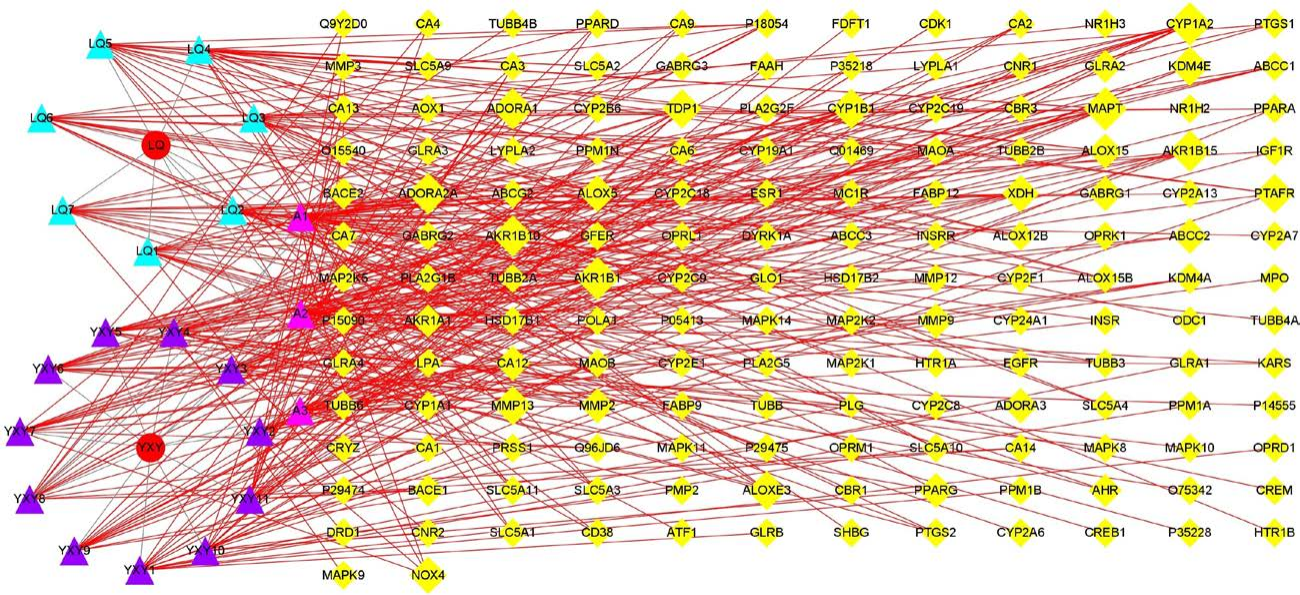
Compound (GF-FF)-target (CA) network (7 blue triangles are FF active compounds, 11 purple triangles are GF active compounds, 158 yellow diamonds are potential targets, 3 pink triangles are public compounds). CA = cerebral atherosclerosis, FF = forsythiae fructus, GF = Ginkgo Folium.

The screened active compounds were identified by the Swiss Target Prediction system, and 158 target genes were finally identified by removing duplicate values. After importing the compound and target genes into Cytoscape 3.8.0, a target network of compound-targets was constructed. There were 181 nodes (namely, 2 herbs, 23 active compounds, and 156 compound targets) and 369 edges, as shown in Figure 2. The 3 communal compound components of GF and FF were kaempferol, luteolin, and quercetin. In the compound-target network, the sizes of the potential targets represent different degrees of closeness to the active compounds.

### 3.2. Identification of cross-target genes

**Figure 3.**
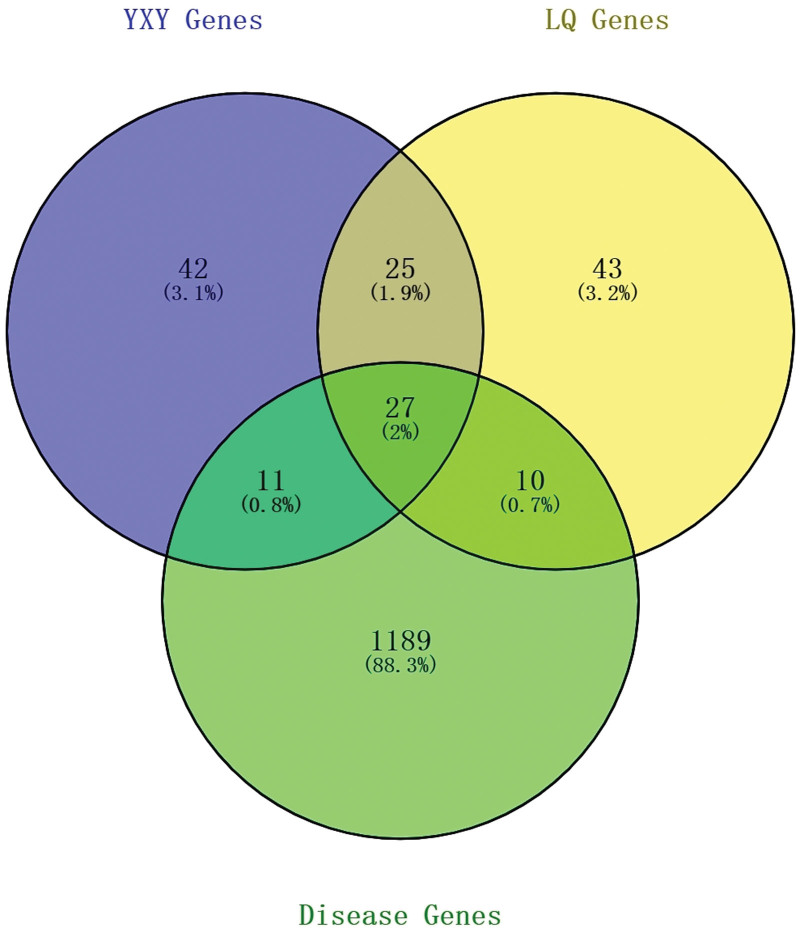
Venn diagram of GF and FF gene targets. FF = forsythiae fructus, GF = Ginkgo Folium.

The relationship between CA and GF-FF was expressed according to the scores of target genes in the above target network construction mapped to the target genes of CA, respectively. In total, 48 target genes were identified to regulate the extent of CA and were regulated by GF-FF drug components, as shown in Table 2 and Figure 3. The results showed that GF-FF regulates slightly more targets of disease.

**Table 2:** GF, FF candidate compound target information.

Number	Target	UniPort*ID	Number	Target	UniPort*ID
1	ABCC1	P33527	25	MAPK8	P45983
2	ABCC2	Q92887	26	MAPK9	P45984
3	ADORA1	P30542	27	MAPT	P10636
4	ADORA2A	P29274	28	MMP12	P39900
5	AHR	P35869	29	MMP13	P45452
6	AKR1B1	P15121	30	MMP2	P08253
7	ALOX15	P16050	31	MMP3	P08254
8	ALOX5	P09917	32	MMP9	P14780
9	CREB1	P16220	33	MPO	P05164
10	CYP19A1	P11511	34	NOX4	Q9NPH5
11	CYP1A1	P04798	35	NR1H2	P55055
12	CYP2B6	P20813	36	NR1H3	Q13133
13	CYP2C19	P33261	37	PLA2G1B	P04054
14	CYP2C9	P11712	38	PLG	P00747
15	CYP2E1	P05181	39	PPARA	Q07869
16	EGFR	P00533	40	PPARD	Q03181
17	ESR1	P03372	41	PPARG	P37231
18	FABP12	A6NFH5	42	PRSS1	P07477
19	FDFT1	P37268	43	PTAFR	P25105
20	IGF1R	P08069	44	PTGS1	P23219
21	INSR	P06213	45	PTGS2	P35354
22	LPA	P08519	46	SHBG	P04278
23	MAPK10	P53779	47	SLC5A1	P13866
24	MAPK14	Q16539	48	XDH	P47989

FF = Forsythiae Fructus, GF = Ginkgo Folium, NO = nitric oxide.

### 3.3. PPI network construction and analysis

**Figure 4.**
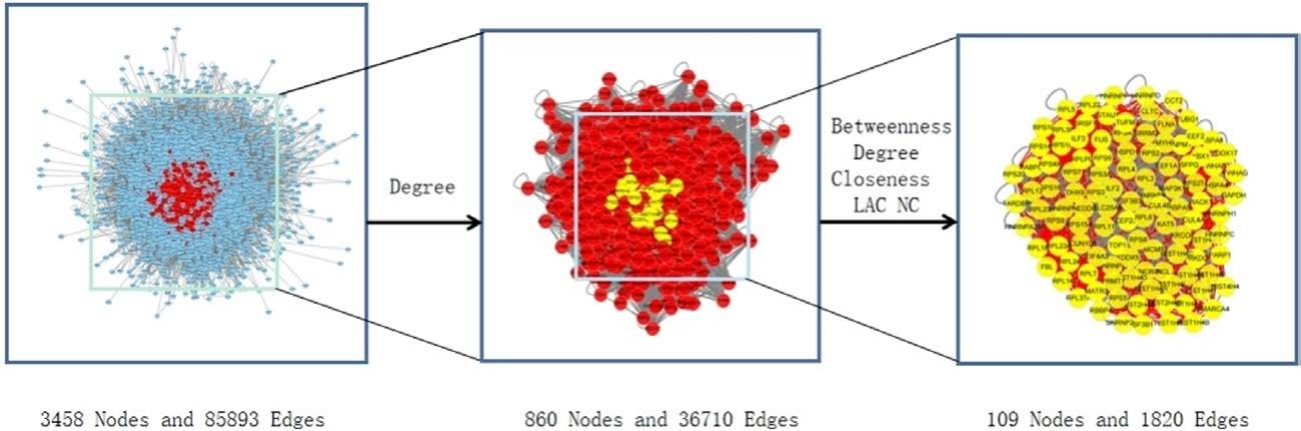
PPI network of GF-FF against CA (Nodes, targets; edges, interaction among targets. Filtered by betweenness, closeness, degree, lAC, nC). CA = cerebral atherosclerosis, FF = forsythiae fructus, GF = Ginkgo Folium, PPI = protein-protein interaction.

To visualize the gene target regulation network, we performed PPI network construction for composite targets–CA disease targets–other human protein targets. We combined the Genecards database to identify the disease targets regulated by cerebral atherosclerosis, and imported into Cytoscape 3.8.0 to intersect with the traditional chinese medicine targets, using 2 screening processes: the first with the criterion “> or equal to 2 times the degree value” and the second with the criterion “> or equal to degree, betweenness, closeness, LAC, neighborhood connectivity median value.” The final PPI network was constructed with 109 nodes and 1820 edges as shown in Figure 4.

We imported the intersected targets into STRING, removed the free protein targets, and obtained 12 core protein targets by topological analysis with the indexes “degree,” “betweenness centrality,” and “closeness centrality” as shown in Table 3.

**Table 3:** 12 PPI network important protein targets.

Name	Degree	Betweenness centrality	Closeness centrality
EGFR	21	0.150511987	0.642857143
CYP2E1	17	0.024917708	0.569620253
CREB1	15	0.070794432	0.592105263
CYP19A1	12	0.02491894	0.569620253
PTGS2	29	0.158894291	0.737704918
PPARG	25	0.127371444	0.671641791
PPARA	22	0.159058251	0.661764706
ESR1	18	0.056249907	0.584415584
MMP9	18	0.053149448	0.616438356
MAPK14	15	0.038993119	0.592105263
MAPK8	14	0.029150203	0.592105263
PLG	12	0.026285406	0.548780488

PPI = protein-protein interaction.

### 3.4. GO and KEGG pathway enrichment analyses

**Figure 5.**
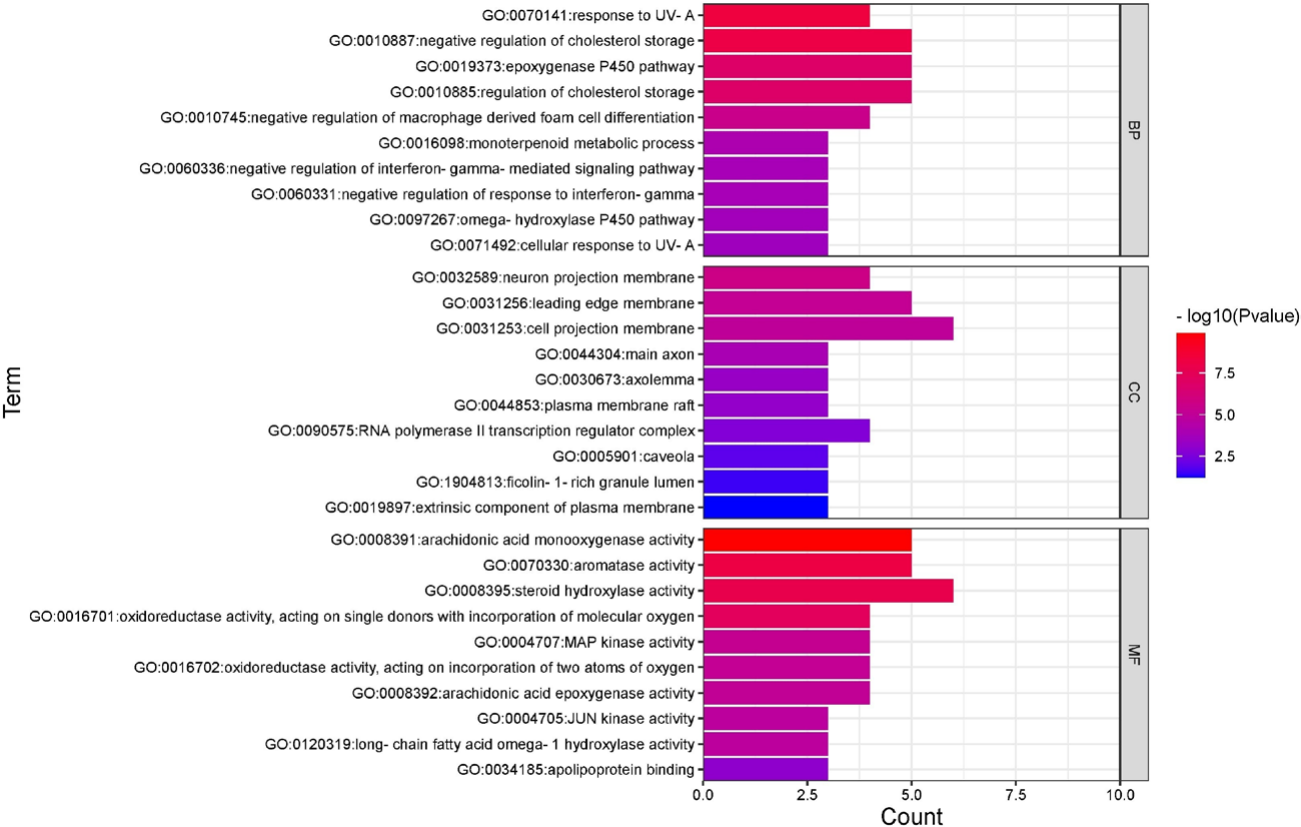
GO analysis for the core targets. GO = gene ontology.

The 12 core protein targets of GF-FF were introduced into the Metascape system for GO and KEGG enrichment analysis, and the species was set to “*Homo sapiens*.” We extracted 10 items in each functional region based on the enrichment values and found that they were related to “monoterpenoid metabolic process,” “regulation of cholesterol storage,” “negative regulation of macrophage derived foam cell differentiation,” “arachidonic acid epoxygenase activity,” “oxidoreductase activity,” and other biological processes. We finally imported the data into the Bioinformatics system for visual analysis as shown in Figure 5.

**Figure 6.**
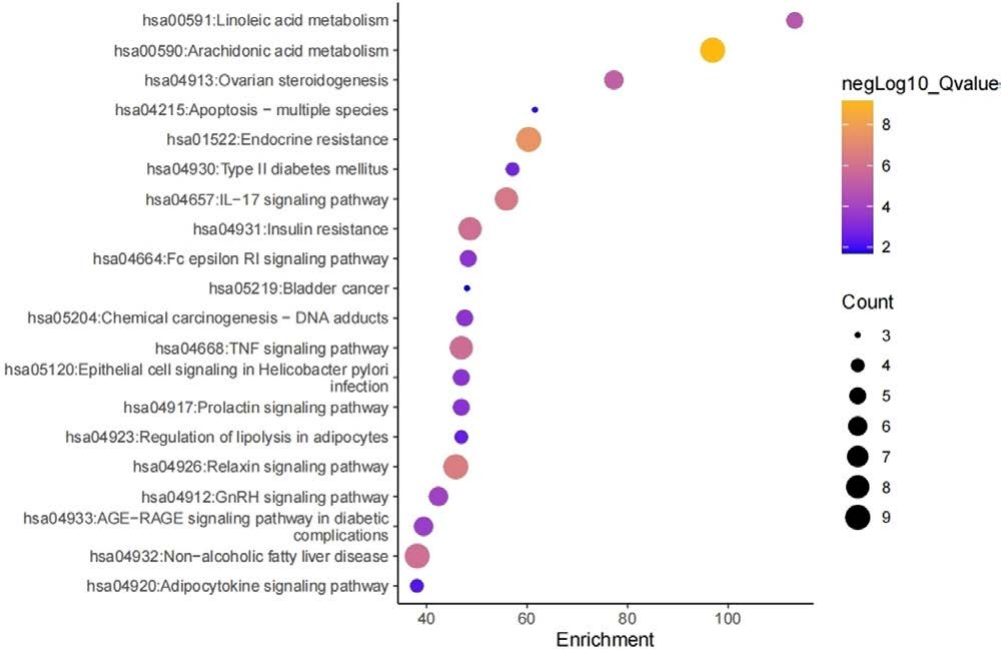
KEGG enrichment analysis for the protein targets. KEGG = kyoto encyclopedia of genes and genomes.

We performed KEGG enrichment analysis showing the top 20 pathways, including “arachidonic acid metabolism,” “apoptosis–multiple species,” “endocrine resistance,” “insulin resistance,” “regulation of lipolysis in adipocytes,” “IL-17 signaling pathway,” and “relaxin signaling pathway.” The imageGP platform was used for visualization and analysis, as shown in Figure 6.

### 3.5. Disease protein-target regulatory pathway analysis

**Figure 7.**
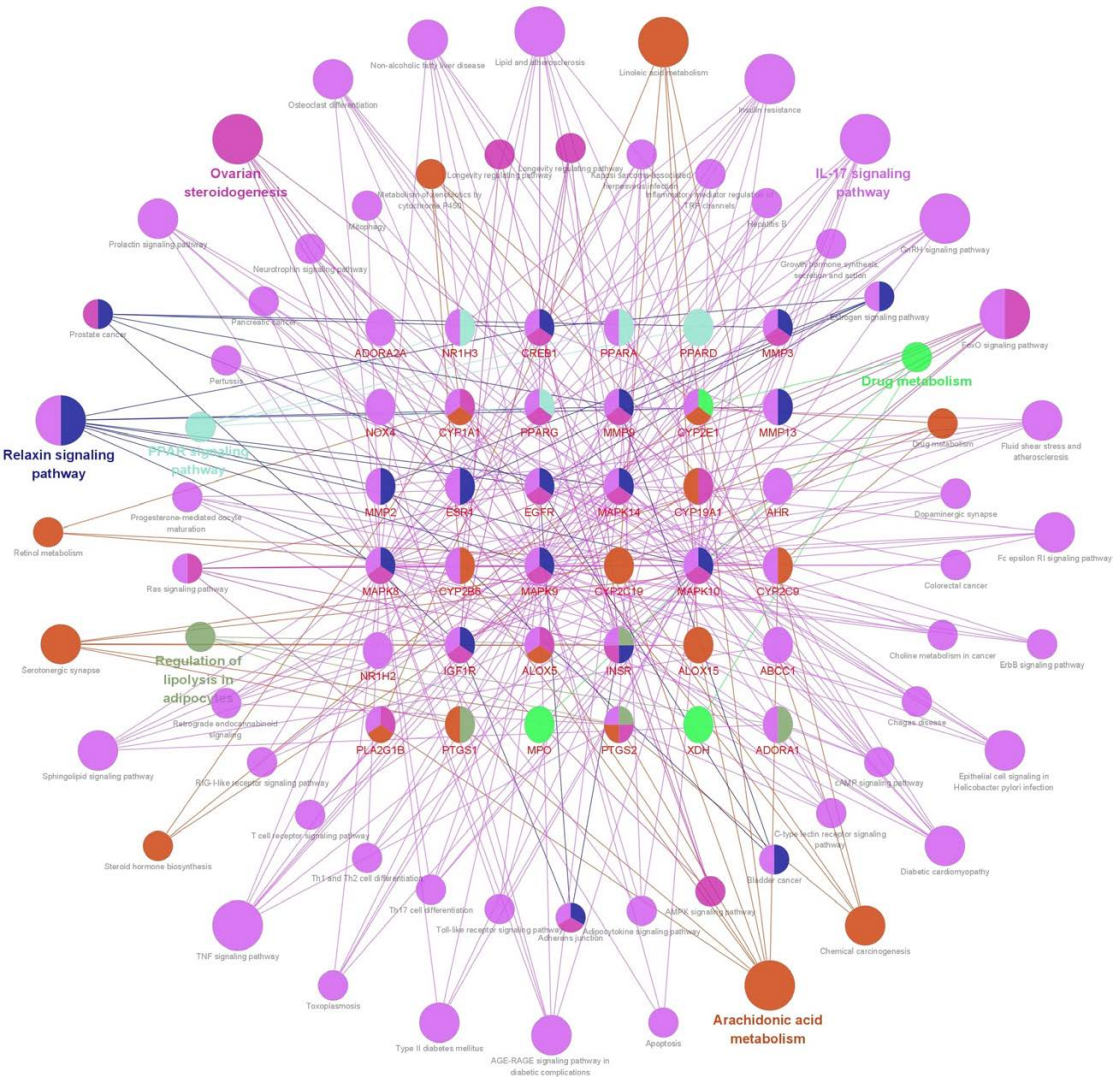
Construction of a disease-protein-target pathway network.

We imported proteins from the STRING database after removing free targets into Cytoscape 3.8.0 software together with the pathways and used its plug-in ClueGo 2.5.8 to visualize the protein-target-gene pathway network. The results showed that “IL-17 signaling pathway,” “relaxin signaling pathway,” “TNF signaling pathway,” “arachidonic acid metabolism,” “type II diabetes mellitus,” “regulation of lipolysis in adipocytes,” “AGE-RAGE signaling pathway in diabetic complications,” “linoleic acid metabolism,” and other pathways played an important mechanism in regulating apoptosis and anti-inflammatory and anti-lipid effects, and providing vascular protection, as shown in Figure 7.

### 3.6. Compound-target docking studies

**Figure 8.**
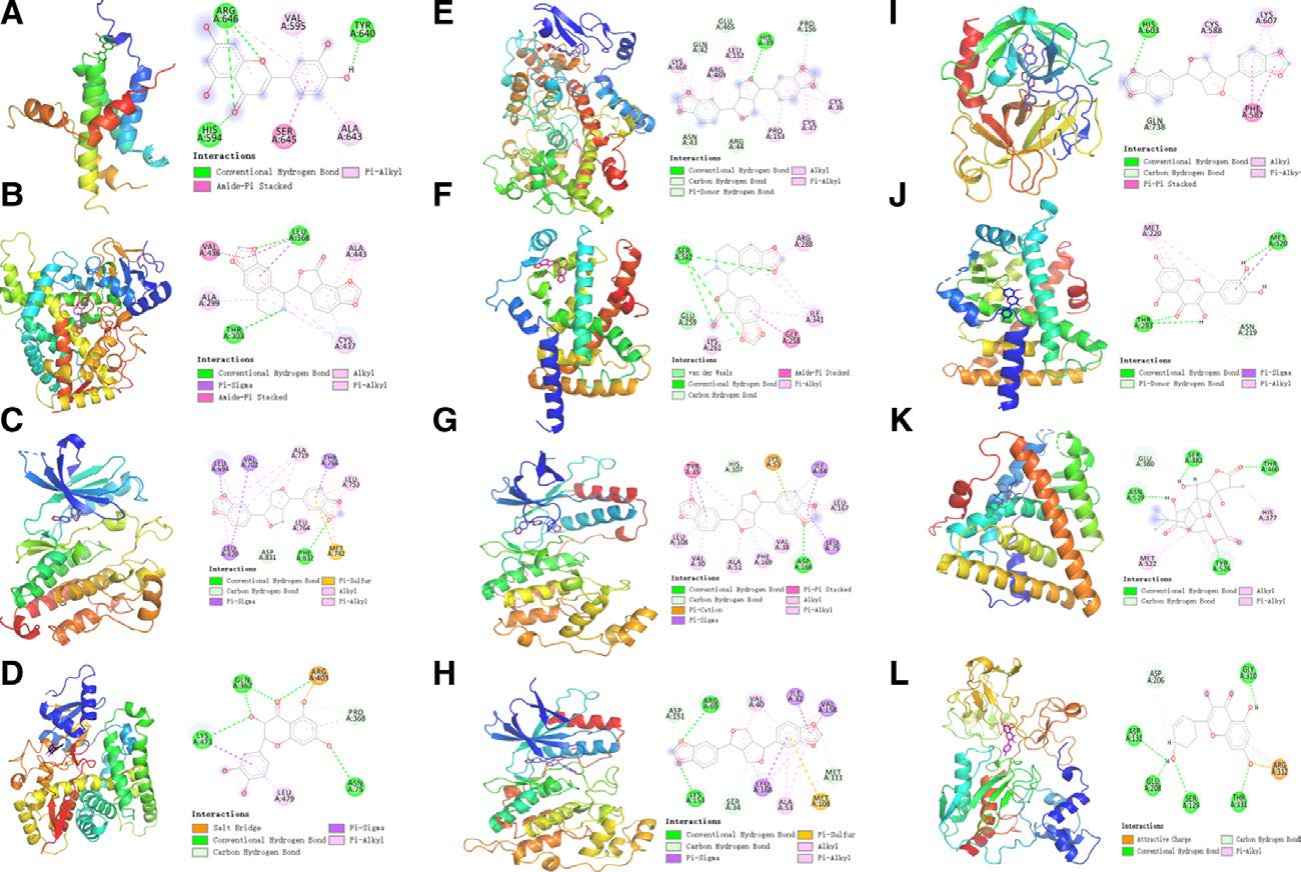
Molecular docking optimal binding diagram.

Based on the network pharmacology results, we screened 6 important compounds – kaempferol, luteolin, quercetin, sesamin, ginkgolide B, and bicuculline – for molecular docking with the core protein targets to further investigate the interactions. Binding affinities below -5 kcal/mol indicated good interactions.^[38]^ The results showed that the active compounds had good binding and reliable interactions with the core protein targets, as shown in Table 4, and the visualized conformation of the compound target proteins is shown in Figure 8.

**Table 4:** Six Compounds from GF-FF for Virtual Docking of CA Targets.

Compound Target	kaempferol	luteolin	bicuculline	quercetin	sesamin	ginkgolide B
EGFR	−8.7	−9.0	−10.1	−8.8	−10.6	−7.5
CYP2E1	−8.8	−8.8	−11.0	−8.9	−9.6	−8.2
CREB1	−6.6	−8.0	−7.9	−6.7	−7.9	−7.6
CYP19A1	−7.8	−8.4	−8.1	−9.9	−9.1	−9.0
PTGS2	−9.3	−9.4	−9.6	−8.2	−11.0	−8.0
PPARG	−7.1	−7.4	−8.5	−7.3	−8.5	−7.1
PPARA	−8.3	−7.8	−7.8	−8.9	−8.9	−7.6
ESR1	−8.4	−8.3	−8.9	−8.2	−6.8	−9.0
MMP9	−9.3	−8.0	−7.5	−8.2	−8.6	−7.9
MAPK14	−8.6	−9.3	−8.9	−8.5	−10.8	−8.1
MAPK8	−7.8	−8.3	−9.1	−8.2	−9.7	−7.1
PLG	−7.5	−7.9	−8.7	−8.0	−9.0	−7.1

CA = cerebral atherosclerosis, GF-FF = Ginkgo Folium-Forsythiae Fructus.

The results showed that sesamin could bind to EGFR, PTGS2, MAPK14, MAPK8, and PLG (Fig. 8A, E, J–L). Figure 8A shows that sesamin can form hydrogen bonding, hydrophobic, and van der Waals force interactions with EGFR protein sites, with strong hydrophobic interactions to amino acids LEU-694 and LEU-820, and strong hydrogen bonding interactions with amino acid PHE-32. Sesamin can form hydrogen bonding, conjugation, and hydrophobic interactions with PTGS2, PPARA, and PLG protein targets (Fig. 8E, L). Figure 8J indicates the presence of hydrogen bonds (ASP-168) as well as hydrophobic interactions (TYR-35, LEU-108, VAL-30) between sesamin and MAPK14 protein targets that allow the small molecule complexes to be active. Figure 8K indicates that sesamin formed strong hydrophobic interactions with amino acids VAL-40, ILE-32, and LEU-168 at the MAPK8 protein locus, and in addition, formed strong hydrogen bonding interactions with LYS-153 and ARG-69.

The results showed that quercetin could bind efficiently to CYP19A1 and PPARA (Fig. 8D, G). Figure 8D shows that quercetin can form hydrogen bonding, hydrophobic, and van der Waals force interactions with CYP19A1 protein sites, including strong hydrophobic interactions with amino acids PRO-368 and LEU-479, and strong hydrogen bonding interactions with amino acids GLN-367 and ASN-75. Figure 8E shows that quercetin has hydrogen bonds and hydrophobic interactions with PPARA protein targets.

The results showed that bicuculline was able to bind well to CYP2E1 and PPARG (Fig. 8B, F). Figure 8B shows that bicuculline can form various interactions with CYP2E1 protein sites, with strong hydrophobic interactions with amino acids VAL-436 and ALA-299, and strong hydrogen bonding interactions with LEU-368 and THR-303. Figure 8F shows that bicuculline is able to form hydrogen bonding, conjugation, and hydrophobic interactions with PPARG protein targets, and these interactions can effectively promote the formation of stable complexes between small molecules and proteins.

The results showed that luteolin, kaempferol, and ginkgolide B had good binding with CREB1, MMP9, and ESR1, respectively (Fig. 8C, I, H). Figure 8C shows the amino acid residues of luteolin interacting with the active site of CREB1 protein, including at ARG-646 and HIS-594. Figure 8I indicates that kaempferol forms strong hydrogen bonding interactions with amino acids ASP-131 and ARG-312 of the MMP9 protein locus. Figure 8H shows that the amino acid residues of ginkgolide B interacting with the active site of ESR1 protein are SER-381 and THR-460. Thus, luteolin, kaempferol, and ginkgolide B have been shown to be important in stabilizing small molecules in protein cavities.

In summary, sesamin binds well to 5 core targets: EGFR, PTGS2, MAPK14, PPARA, and MAPK8. Quercetin binds well to 2 protein targets, CYP19A1 and PPARA. Bicuculline binds well to 2 core proteins, CYP2E1 and PPARG. Kaempferol, luteolin, and ginkgolide bind well to CREB1, MMP9, and ESR1, respectively. Therefore, we believe that the 6 core compounds of GF-FF may play a key role in the treatment of CA.

## 4. Discussion

According to the Chinese Pharmacopoeia,^[39]^ GF has pharmacological effects, including blood stasis activating, free radical scavenging, anti-myocardial ischemia, hypolipidemic, hypoglycemic, anti-inflammatory and antioxidant, and vascular and neurological function protective effects, and FF has pharmacological effects, including antibacterial and antiviral, anti-inflammatory, antioxidant, and apoptosis-inhibiting effects.

CA is a chronic cerebrovascular disease characterized by a metabolic syndrome of lipid accumulation, elevated cholesterol, and insulin resistance,^[2,40,41]^ accompanied by apoptotic necrosis leading to local vascular stenosis and embolism,^[4]^ and local inflammatory-oxidative responses throughout the development and progression of CA. Currently, the typical event in the mechanism of CA is that oxidative low-density lipoprotein (ox-LDL) is chemotactic to monocytes, upregulating them to produce monocyte colony-stimulating factor and monocyte chemoattractant protein.^[42]^ Activation of monocyte components produces inflammatory factors that induce oxidation of LDL, and oxidative LDL leads to CA formation and exacerbation.^[43,44]^

A total of 23 active compounds were obtained through TCMSP and SwissADME clinical pharmacological screening for 158 compound targets, by predicting 6 components – sesamin, quercetin, bicuculline, kaempferol, luteolin, and ginkgolide B – as core compounds, among which quercetin, kaempferol, and luteolin are known compounds of GF-FF.

It has been shown that sesamin alters cellular metabolism and mitigates local oxidative and inflammatory changes through receptor catalysis of PPARα/γ, with down-regulation of the SREBP-1/arachidonic acid metabolism signaling pathway and MAPK/NF-κB/TNFα/ extracellular signal-regulated kinase (ERK) 1/2 signaling pathway.^[18,45]^ Quercetin inhibits cellular oxidation and disrupts inflammatory transmission by stimulating ERK1/2 and P38MAPK factors to improve β-cell function and thus modulate signaling pathways such as the AGE-RAGE/endocrine resistance pathway.^[46]^ GABA(A)R receptors in bicuculline alleviate β-cell dysfunction by promoting the phosphoinositide 3-kinase (PI3K)/Akt signaling pathway downstream to exert insulin resistance and hypotensive and antilipidemic effects. Kaempferol can have a protective effect on brain neurology by reducing PTGS2 and IL-6 levels to inhibit neuroinflammation, reactive oxygen species, and neuronal apoptosis.^[47]^

Luteolin acts on the cyclic adenosine monophosphate signaling pathway by inhibiting CREB1 expression and downregulates mRNA expression to affect insulin expression and inhibit apoptotic effects, Ginkgolide B enhances the expression of ERK1/2/p-EGFR signaling pathway by activating EGFR/Akt trans receptors,^[48,49]^ and also alters lipid metabolism and thus protects neurological function by inhibiting IL-1α/β expression. Taken together, it is speculated that the use of GF-FF for the treatment of cerebral atherosclerosis should be a multi-component, multi-target interaction, and the mechanisms of action should be studied more in depth.

In this study, EGFR, CREB1, PTGS2, PPARG, PPARA, MMP9, MAPK8, and MAPK14 were identified as core protein targets for the treatment of CA. Reducing the expression of PPARα, MMP9, and IL-1β proteins, elevate MAPK levels, inhibit reactive oxygen specie production, and enhance nitric oxide bioactivity in blood vessels, thereby ameliorate endothelial disorders and hypertension, reducing the inflammatory response, and altering the progression of cerebral atherosclerosis and thrombosis.^[50]^ Activation of EGFR protein reverse transcription can stimulate the PI3K pathway and ERK regulatory enzyme production, protecting cells from apoptosis.^[49]^ CREB1 protein activation turns on the transcription of downstream genes as a pathway of cellular metabolic activity, accelerating glucose uptake and maintaining the stability of the body’s internal environment.^[51]^

GO analysis showed that GF-FF is involved in biological processes such as cholesterol storage regulation, negative regulation of macrophage-derived foam cell differentiation, arachidonic acid cyclooxygenase activity, and oxidase activity. Macrophage differentiation into lipid-rich foam cells, a hallmark of the early stages of cerebral atherosclerosis, inhibits oxLDL-mediated phagocytic foam cell formation and reduces the size of atherosclerotic lesions by downregulating LOX-1 and mRNA protein expression through the AMPK and TNF-α regulatory pathways.^[52]^ Regarding the process of arachidonic acid and peroxidase regulation, the ∆ 5 desaturated acid output profile affects n-6 unsaturated fatty acids, which in turn regulates arachidonic acid production; sesamin increases peroxidase mRNA levels, thereby activating PPARα and PTGS2 pathway regulation for the treatment of hypertension and lipid oxidation.^[45,53]^ Therefore, we speculate that GF-FF regulation of CA may be related to the above biological processes.

KEGG pathway analysis showed that GF-FF treats CA pathways, including “arachidonic acid metabolism,” “apoptosis–multiple species,” “endocrine resistance,” “insulin resistance,” “regulation of lipolysis in adipocytes,” “IL-17 signaling pathway,” “AGE-RAGE signaling pathway in diabetic complications,” “TNF signaling pathway,” and “relaxin signaling pathway.” The TNF signaling pathway has anti-inflammatory effects and regulates apoptosis and other protective neurological functions by activating the TNF-RI protein pathway through TNF-α, leading to the expression of the transcription factor NF-κB, producing the cytokines IL-1α/β and resisting cellular inflammation and apoptotic processes.^[54,55]^ The IL-17 signaling pathway plays a major role in cellular metabolism and anti-inflammation in the body, and has a significant role in fighting inflammation by inducing the expression of cytokines and chemokines targeting NF-κB and MAPK through the IL receptor family.^[56]^ In summary, GF-FF herbal compounds modulate numerous pathways such as AGE-RAGE, TNF, and IL-17, and therefore are considered to treat CA. We speculate that GF-FF could play an important role in the treatment of CA.

The present study still has some limitations, we used only modern bioinformatics methods to explore the effects of GF-FF in the treatment of CA by using network pharmacology and molecular docking. Firstly, data from online databases are based on reviewed and predicted data, and unconfirmed and unrecorded data were not included in our study. Secondly, for the 23 compounds as well as the 6 core compounds identified here, quantitative studies are incomplete, and future studies should be done on the content of the components. Thirdly, although sesamin, quercetin, bicuculline, kaempferol, luteolin, and ginkgolide B were identified as important compounds, this does not fully represent GF-FF, and therefore pharmacodynamic and molecular biology experiments need to be considered to further investigate our results. Therefore, the potential mechanism of action of GF-FF for the treatment of CA has not yet been explained and confirmed, and we believe that this topic has great research potential and application value.

## 5. Conclusion

In this study, sesamin, quercetin, bicuculline, kaempferol, luteolin, and ginkgolide B were identified as important herbal compounds, and EGFR, CREB1, PTGS2, PPARG, PPARA, MMP9, MAPK8, and MAPK14 were identified as major protein targets for the treatment of CA. Molecular docking showed that these compounds and their targets have good binding effects. GF-FF may exert therapeutic effects through the AGE-RAGE, TNF, IL-17, and MAPK signaling pathways. In addition, our study approach may further elucidate the mechanism of GF-FF for the treatment of CA.

## Author contributions

**Data curation:** Jinfei Zhang.

**Formal analysis:** Jinfei Zhang, Jiqin Tang.

**Methodology:** Jinfei Zhang, Jiqin Tang, Chuntao Yang, Guoxiu Zu.

**Investigation:** Chuntao Yang, Guoxiu Zu.

**Funding acquisition:** Jiqin Tang.

**Writing – original draft:** Jinfei Zhang, Jiqin Tang.

**Writing – review & editing:** Jinfei Zhang, Jialin Gai, Hengqin Ma.
